# Allocating vaccines to remote and on-site workers in the tradable sector

**DOI:** 10.1038/s41598-022-08043-0

**Published:** 2022-03-08

**Authors:** László Czaller, Gergő Tóth, Balázs Lengyel

**Affiliations:** 1 Agglomeration and Social Networks Lendület Research Group, ELKH Centre for Economic and Regional Studies, Budapest, 1097 Hungary; 2 Spatial Dynamics Lab, University College Dublin, Belfield, Dublin 4, Ireland; 3 Laboratory for Networks, Technology and Innovation, Corvinus University of Budapest, Budapest, 1093 Hungary

**Keywords:** Viral infection, Socioeconomic scenarios

## Abstract

Vaccination may be the solution to the pandemic-induced health crisis, but the allocation of vaccines is a complex task in which ethical, economic and social considerations are important. The biggest challenge is to use the limited number of vaccines available in a way that protects vulnerable groups, prevents further spread of infection, and reduces economic uncertainty. We argue that once the vaccination of healthcare workers and the most vulnerable groups has been completed, prioritizing the vaccination of on-site workers is important not only to slow the spread of the infection, but also to ensure the smooth running of economic production. We propose a simple economic model where remote and on-site workers are complementary to each other in the short run, thus a negative shock to the supply of either one may decrease the demand for the other, leading to unemployment. By illustrating the model using pre-Covid employment data from Sweden and Hungary, we show that the optimal vaccine allocation between remote and on-site workers in the tradable sector should be based on different proportions depending on the relative infection risk of on-site workers and the degree of vaccine availability. As long as the number of vaccines is limited and on-site workers are at higher risk of infection, they should be preferred in general. However, as more vaccines become available, countries like Sweden, where the share of occupations that can be done remotely is higher shall start immunize remote workers. In Hungary, where on-site work is dominant in the tradable sector, continued vaccination of on-site workers is more beneficial.

## Introduction

Restricting individual mobility and introducing different social distancing measures can slow down the spread of respiratory viruses^1,2^, but they come with enormous economic consequences^3^. During the first waves of the COVID-19 pandemic, the unprecedented fall in consumer demand has led to many job losses and disruptions in production chains^4–6^, through which the negative effects of preventive interventions have spread throughout the economy^7,8^. To strike a balance between the epidemiological benefits and economic costs of mobility restrictions, commuting to work was allowed in most countries despite the fact that work-related virus transmission had been found to be considerable in early COVID-19 outbreaks^9^. People were advised to work from home where possible and employers were encouraged to consider ’hybrid’ models with home and on-site working. However, the option of working from home is not equally available to all. Remote work is a realistic option in occupations where physical presence and personal contacts with others are not prerequisites for productive work^3,10,11^. Those who cannot work from home have to face higher risks of infection in order to keep their jobs^12,13^. This makes on-site workers a vulnerable socioeconomic group that has an impact on virus transmission.

The development of vaccines has created a new situation and the question governments must quickly answer is how to allocate the limited number of early vaccines to curb infection and ease the restrictions. The consensus is that vaccine allocation must be optimized to save lives and must favor vulnerable groups^14,15^. However, some argue that vaccination plans should favor those who come into contact with many people and carry more infections^16–19^. Despite their importance^20^, economic rationals are almost completely ignored in this discussion. One exception is Cakmakli et al.^21^ who illustrates that an ethical distribution of vaccines across countries^22^ can pay off in functioning global production and supply chains. Yet, the notion that risk of infection between remote and on-site workers may differ is still missing from this discourse and left out from vaccination strategies in most countries. The primary aim of this paper is to highlight the economic rationale behind the vaccination of on-site workers, especially in the tradable goods sector, which has been the least restricted industry during the pandemic. While a high vaccination rate for workers in non-teleworkable occupations may be justified from an epidemiological point of view (due to their higher exposure to infection), favoring on-site workers also serves to reduce unemployment.

We consider a simple model in which workers perform two tasks to produce goods. The important difference between the two tasks is that while one can be performed from home, the other can only be performed on site. The central element of our model is that in the short run, firms can adjust production by changing the number of employees in both tasks, but they cannot change the production technology that determines the exact proportion of task inputs. Hence, in the short run, the two types of tasks are perfect complements and must be used in fixed proportions. Under such conditions, if the infection risks of remote and on-site workers differ, mass infection among on-site workers will reduce the demand for workers who can perform tasks from home, which may lead to excess unemployment. Hence, although remote workers are presumably less exposed to infection, they also bear the burden of job loss due to the complementarity of different tasks.

Our analysis is based on the assumption that the infection risk differs among occupations in the short run. Recent empirical studies show that occupational characteristics, such as interfacing with the public, working indoors and being in close quarters with others put on-site workers at high risk for infection, while those working in isolation face lower risks^23^. This is consistent with the finding that excess mortality tends to be higher in occupation groups where remote work is less feasible (e.g. food and agriculture, manufacturing, transport and logistics)^24^, even after controlling for individual characteristics such as race, ethnicity, place of residence or income^25^. These results suggest that, despite the majority of the population is expected to contract the infection in the long run, there may be significant differences between remote and on-site workers in terms of infection probability over a shorter time period. We build this possibility into the model by assigning different infection risks to remote and on-site workers. These risk parameters give the expected proportion of infected workers in job types within a given time period. Although this approach is undoubtedly abstract and differs from those used in SIR models, it makes it easier to model how short run task complementarity contributes to the rise in unemployment during the pandemic and helps to make straightforward claims about how vaccines should be distributed between remote and on-site workers in order to minimize unemployment.

The model focuses on industries that produce tradable goods (i.e. agriculture, manufacturing, electricity, mining and quarrying) because it intentionally abstracts out a number of factors that have been proven to contribute to the rise of unemployment in non-tradable services during the pandemic. Such factors are the restrictions imposed by governments to curb infections, and changes in consumption habits for fear of infection^6,26^. These effects were probably most pronounced in the non-tradable sector, where restrictions made it impossible to carry out certain activities (i.e. accommodation and food services, arts, entertainment and recreation etc.), and where consumption demand was substantially reduced by fear of infection (i.e. retail, personal services, public administration)^6^. Hence, the economic mechanism we consider in the paper is relatively less important in the non-tradable sector. However, in the tradable sector, where production is not necessarily for final consumption and sales do not require the physical presence of the consumer (because they mostly happen through retail and wholesale), short-run adjustment decisions of firms may play a relatively greater role in explaining the rise in unemployment.

We illustrate the model using pre-Covid employment data for the tradable sector of Hungary and Sweden. According to the model, if the amount of vaccines available is enough for a small fraction of workers in the tradable sector, a larger proportion of vaccines should be given to on-site workers in Hungary than in Sweden to keep unemployment to a minimum. However, as more vaccines become available, immunization of remote workers is becoming increasingly important in Sweden, especially if there is a small difference in the infection risk between remote and on-site workers. However, on-site workers should be preferred in case their risk of infection is much higher than that of remote workers. Moreover, in countries like Hungary, where the tradable sector relies on mostly non-teleworkable tasks, continued vaccination of on-site workers is more beneficial.

The purpose of the paper is not to make specific recommendations on vaccine distribution, but to highlight the economic rationale of distinguishing between remote and on-site workers in the later phases of vaccination schedules when it comes to immunizing non-essential (and non-vulnerable) workers.

## Model

### Basic setup

Consider a sector, where competitive firms produce a single tradable good by combining two types of tasks: (1) teleworkable tasks that can be done from home, and (2) non-teleworkable tasks that must be performed on-site^3,10^. Depending on who performs which task, we distinguish between two types of workers. Those who perform teleworkable tasks are referred to as remote workers (*r*), while those who perform tasks requiring physical presence are referred to as on-site workers (*s*). Suppose that workers are able to perform both tasks with unit productivity but once they are trained for one of the tasks, they cannot switch to the other. Since firms are unable to change technology or redesign business organization in the short-run, worker proportions remain fixed. This implies that the production function is Leontief,

1 $$\begin{aligned} Y=\min \left( \alpha _r L_r, \alpha _s L_s \right) , \end{aligned}$$

where $L_r$ refers to the number of remote workers, $L_s$ refers to the number of on-site workers performing non-teleworkable tasks, $\alpha _r$ and $\alpha _s$ are unit input requirements and *Y* is output. Taking labor supply, wages and unit input requirements as given, firms optimize $L_r$ and $L_s$ in order to maximize profit that implies

2 $$\begin{aligned} Y = \alpha _r L_r = \alpha _s L_s. \end{aligned}$$

The pandemic starts after firms have optimized labor usage. Once the outbreak occurs, the government obligates firms to send workers performing teleworkable tasks to home-office. As a consequence, the probability of becoming infected will be decreased for remote workers, while the exposure of on-site workers performing non-teleworkable tasks will remain unaffected. Formally, let $\beta _i$ be the short-run probability of infection for worker type $i \in \{r,s\}$ such that $\beta _r \le \beta _s$. Opportunities for remote work, however, come with a price. Although working from home benefits employees by eliminating their daily commutes it may decrease their productivity by making communication, negotiation and instructing more cumbersome, and also by increasing reaction time in decision situations^3,27^. Thus, we assume that the productivity of remote workers decreases to $\gamma \in (0,1)$ as long as they work from home.

Production decreases during the pandemic because effective labor in both tasks deviate negatively from optimal amounts. Without any available vaccines the supply of effective labor reduces to

$$\begin{aligned} \bar{L}_s = (1 - \beta _s) L_s \end{aligned}$$

and

$$\begin{aligned} \bar{L}_r = (1 - \beta _r) \gamma L_r. \end{aligned}$$

Since tasks are perfect complements, reduction of effective labor in one task decreases labor demand in the other task and workers become unemployed. Suppose that $\beta _s > 1- (1-\beta _r)\gamma$ holds, so $\beta _s$ reduces the supply of labor in non-teleworkable tasks to a greater extent than $\beta _r$ and $\gamma$ decrease labor supply in teleworkable tasks. In such cases, $\left[ (1-\beta _r) \gamma - (1-\beta _s ) \right] L_r$ healthy remote workers will be unnecessary for production. However, if $\beta _s < 1- (1-\beta _r)\gamma$, exactly $\left[ (1-\beta _s) - (1-\beta _r)\gamma \right] L_r$ on-site workers will become redundant. Hence, without vaccines, unemployment will inevitably rise despite the extensive spread of home office possibilities.

### Optimal vaccine allocation

Now suppose that a social planner distributes *V* amount of vaccines across *L* workers in the sector. Vaccination of the workforce has two effects: First, it immunizes workers and therefore increases the amount of effective labor, and second, it makes social distancing among vaccinated workers unnecessary. After getting the vaccine, remote workers performing teleworkable tasks restore their full productivity because they are allowed to go back to the office. Vaccines are scarce and not all workers can be immunized ($V \ll L$). Therefore, the social planner aims to distribute the vaccines to minimize job losses of healthy workers. Note that the problem of minimizing unemployment among healthy workers and maximizing output provides similar solutions.

We quantify the share of vaccines that should be allocated to a certain type of worker. Let $v_i$ be the number of immunized workers $i \in \left\{ r,s \right\}$. Assuming that all vaccines are used, $\sum _{i \in (r,s)} v_i = V$ and every worker accepts the vaccine, labor supplies can be expressed using $v_s$ only as:

3 $$\begin{aligned} \bar{L}_s = (1-\beta _s)L_s + \beta _s v_s, \end{aligned}$$

and

4 $$\begin{aligned} \bar{L}_r = (1-\beta _r)\gamma L_r + (1 - \gamma + \beta _r \gamma )(V-v_s). \end{aligned}$$

The social planner’s problem is to minimize job losses arising from task complementarity. This aim can be formalized by the objective function

$$\begin{aligned} \min _{v_s} \left| \alpha _s \bar{L}_s - \alpha _r \bar{L}_r \right| , \quad 0 \le v_s \le V \end{aligned}$$

The global minimum of the objective function is zero which can be found at

5 $$\begin{aligned} \underline{v}_s = \frac{\alpha _r \left[ ( 1-\beta _r)\gamma L_r + (1 - \gamma + \beta _r \gamma )V \right] - (1-\beta _s)\alpha _s L_s}{\alpha _s \beta _s + \alpha _r(1 - \gamma + \beta _r \gamma )}. \end{aligned}$$

Equation (5) can be obtained by substituting $\bar{L}_s$ and $\bar{L}_r$ from Eqs. (3) and (4) into the objective function and then solving it for $v_s$. The threshold defined in the above equation ensures that remote and on-site workers are available in exactly the proportion required by the production technology in Eq. (1). Depending on the value of $\underline{v}_s$, the solution to the social planner’s problem is

$$\begin{aligned} v_s^*= {\left\{ \begin{array}{ll} 0, &{} \text {if }\,\,\underline{v}_s \le 0 \\ \underline{v}_s, &{} \text {if }\,\,0< \underline{v}_s < V\\ V, &{} \text {if }\,\,\underline{v}_s \ge V. \\ \end{array}\right. } \end{aligned}$$

**Figure 1:**
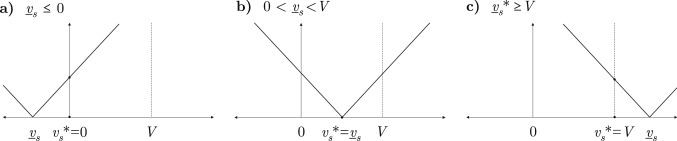
Solutions to the social planner’s problem: (**a**) if $\underline{v}_s \le 0$, all available vaccines should be given to those who can work from home in order to avoid layoffs among on-site workers. (**b**) If $0<\underline{v}_s<V$, then $v_s^*=\underline{v}_s$, which means that there is no unemployment among healthy workers. (**c**) If $\underline{v}_s \ge V$ all vaccines should be given to on-site workers to minimize unemployment among healthy remote workers.

Solutions are depicted in Fig. 1. If $\underline{v}_s \le 0$, we get the corner solution of $v_s^*= 0$, which means that all available vaccines should be given to those who perform teleworkable tasks. In such cases, effective labor in teleworkable tasks determines output and the total amount of on-site labor used for non-teleworkable tasks:

$$\begin{aligned} \tilde{L}_s = (1-\beta _r) \gamma L_s + \frac{\alpha _r}{\alpha _s} (1 - \gamma + \beta _r \gamma ) V. \end{aligned}$$

Substituting $v_s^*=0$ into Eq. (3), and subtracting $\tilde{L}_s$ gives the number of unemployed on-site workers:

$$\begin{aligned} U_s = \left[ (1-\beta _s) - (1-\beta _r)\gamma \right] L_r - \frac{\alpha _r}{\alpha _s} (1 - \gamma + \beta _r \gamma ) V. \end{aligned}$$

Note that, although this vaccine allocation cannot maintain full employment among healthy on-site workers, it still reduces unemployment by $(\alpha _r/\alpha _s) (1 - \gamma + \beta _r \gamma ) V$.

If $\underline{v}_s$ lies within the interval (0, *V*), the social planner is able to find a vaccination plan that provides the optimal proportion of remote and on-site workers. This implies that nobody drops out of work due to the reduction of work capacity in complementary tasks, so $\tilde{L_i} = \bar{L}_i, \forall i \in (r,s)$.

Finally, if $\underline{v}_s \ge V$, all vaccines should be given to on-site workers, $v_s^*= V$, in order to minimize unemployment among workers in teleworkable activities. When on-site workers are the bottleneck of production, employment in teleworkable tasks becomes

$$\begin{aligned} \tilde{L}_r = (1-\beta _r) L_r + \frac{\alpha _s}{\alpha _r} \beta _s V, \end{aligned}$$

which implies

$$\begin{aligned} U_r = \left[ (1-\beta _r) \gamma - (1-\beta _r)\right] L_r - (\alpha _s/\alpha _r) \beta _s V \end{aligned}$$

unemployed remote workers. Compared to the baseline case, this vaccine allocation scheme reduces unemployment in teleworkable tasks by $(\alpha _s/\alpha _r) \beta _s V$.

The optimal allocation of vaccines depends on labor supplies ($L_r$ and $L_s$), the parameters describing the structure of the economy ($\alpha _r$, $\alpha _s$, and $\gamma$), the number of vaccines available (*V*) and the task-specific probabilities of infection ($\beta _r$ and $\beta _s$). It follows that there is no uniform recipe for the distribution of vaccines which derives solely from the characteristics of the economy. By differentiating Eq. (5) with respect to $\beta _s$ we obtain

$$\begin{aligned} \frac{\partial \underline{v}_s}{\partial \beta _s} = \frac{\alpha _s}{\alpha _s \beta _s + \alpha _r(1 - \gamma + \beta _r \gamma )} \left( L_s - \underline{v}_s \right) , \end{aligned}$$

so if the probability of infection for on-site workers increases (e.g. safety regulations are not followed by employees at the workplace), more vaccines should be given to them not just to avoid workplace transmission of the virus but also to minimize redundancy of remote labor. Clearly, if all on-site workers are vaccinated, $\underline{v}_s = L_s$, $\beta _s$ has no further effects on $\underline{v}_s$. In contrast, by differentiating $\underline{v}_s$ with respect to $\beta _r$ we find that an infinitesimal change in the infection risk of remote workers decreases the optimal number of vaccinated on-site workers ($\underline{v}_s$),

$$\begin{aligned} \frac{\partial \underline{v}_s}{\partial \beta _r} = \frac{\alpha _r \gamma }{\alpha _s \beta _s + \alpha _r(1 - \gamma + \beta _r \gamma )} \left( V - L_r - \underline{v}_s \right) , \end{aligned}$$

as long as $\underline{v}_r < L_r$.

## Results

We illustrate the results of our model with numerical simulations. We relate our model to Swedish and Hungarian employment data to examine the extent to which $v_s^*$ varies across economies of different structures and various short-run epidemiological scenarios. Since the model considers a sector where tradable goods are produced, the simulation focuses exclusively on the tradable sectors of both countries. All parameters are based on observational data, except task-specific infection probabilities ($\beta _s$ and $\beta _r$). Since these parameters, as they appear in the model, are difficult to relate to real-life estimates, we consider all combinations of $\beta _s$ and $\beta _r$ parameters where $\beta _s \ge \beta _r$. For a more detailed description of the data, see “Material and methods” section.

**Figure 2:**
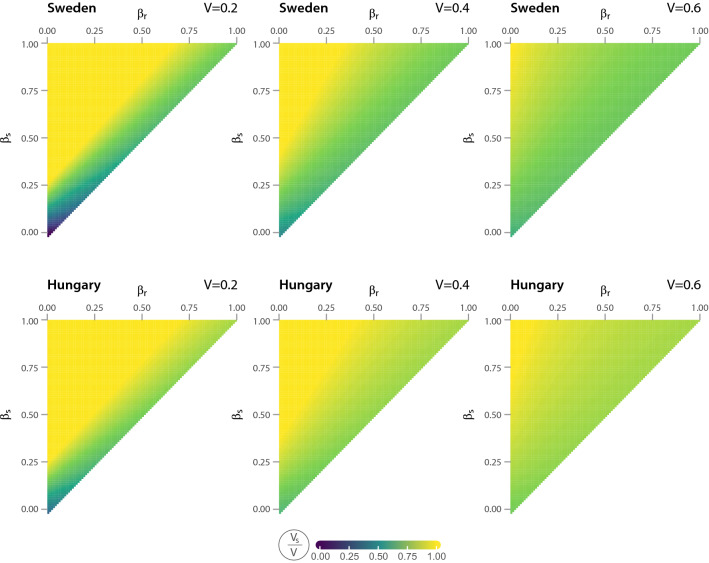
Vaccine allocation by occupation-specific infection risks and available vaccines. Colors represent values of on-site worker fraction in vaccines $v_s$, $\beta _r$ is theoretical infection risk of remote workers, $\beta _s$ is theoretical infection risk of on-site workers. *V*/*L* stand for the share of worker population that can be vaccinated with available vaccines.

Figure 2 depicts the share of vaccines given to on-site workers ($v_s/V$) as a function of $\beta _s$ and $\beta _r$ for two countries with different shares of on-site jobs in the tradable sector: Sweden (high $L_s/L$), and Hungary (low $L_s/L$). While in Sweden almost a quarter of jobs can be done from home in the tradable sector (22.6%), in Hungary the share of teleworkable jobs is only 13%. Each subplot considers three scenarios that represent different levels of vaccine scarcity. For example, the first scenario described by $V/L = 0.2$ means that only 20% of employees receive a vaccine while the scenario of $V/L = 0.6$ describes the case when there is enough vaccines to immunize 60% of the workers. We consider only those parameter combinations where $\beta _s \ge \beta _r$ (above the matrix diagonal) to avoid the cases when the simulation covers unrealistic infection rates (e.g. when remote workers are exposed to the virus, while those working on the site are protected).

**Figure 3:**
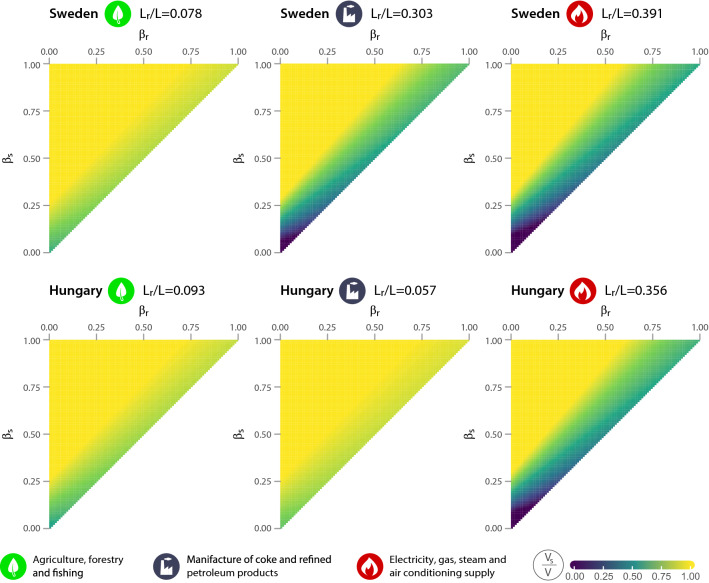
Vaccine allocation by occupation-specific infection risks and available vaccines in selected sectors. The color scale shows the fraction of vaccines given to on-site workers ($v_s/V$), $\beta _r$ and $\beta _s$ are the short-run infection risk of remote and on-site workers respectively and *V*/*L* represents the share of workers that can be vaccinated with available vaccines. $V/L = 0.2$.

Perhaps the most conspicuous pattern of this figure is that in Sweden, where the share of remote workers is high, optimal vaccine allocations favor remote workers for many epidemic scenarios especially when vaccine is a scarce resource and the risk of infection is low in both groups of workers. In such cases, vaccinating remote workers compensates for the productivity loss from teleworking. However, if on-site workers have a much higher chance of infection during waves of virus diffusion, the focus shifts from remote workers to on-site workers. In Hungary, where remote work is less common, high $v_s/V$ ratios can be observed even in some cases where $\beta _r$ is high as well. Such economies will do well if they concentrate on vaccinating on-site workers throughout the pandemic. The differences between countries are blurred when vaccines are widely available. In this case, on-site worker vaccination should exceed remote worker vaccination in most risk scenarios. On this basis, a higher proportion of available vaccines should be given to individuals who, due to the nature of their jobs, cannot work from home.

Given that industries within the tradable sector differ in terms of the proportion of jobs that can be done from home, it is worth looking at the optimal $v_s/V$ ratios at the level of industries. Figure 3 depicts the optimal share of vaccines given to on-site workers as a function of task-specific infection probabilities for a selection of industries in the tradable sector. On the left side of the figure are the $v_s/V$ ratios calculated for agriculture, forestry and fishing. Due to the low prevalence of teleworking (the share of teleworkable jobs is below 10% in both countries), on-site workers receive the vast majority of vaccines in most infection scenarios. However, when looking at the manufacture of coke and refined petroleum products, there are significant differences between the two countries. In Hungary, this industry relies almost exclusively on on-site work ($L_r/L = 0.06$), the share of vaccines given to remote workers is minimal even when $\beta _r$ is high. In Sweden, however, remote work plays a greater role in the production of coke and processed petroleum products; thus, their vaccination should be more intensive in case of small infection probability differences. However, if we look at the subplot to the right of the figure (electricity, gas and steam supply), we can again draw the same conclusions for the two countries. As long as the chances of infection are equally low in both worker groups and the availability of vaccines remains limited, vaccinating remote workers can ensure the smooth flow of industry production during the pandemic. Overall, the extent to which the pandemic affects individual industries within the tradable sector may vary significantly depending on the prevalence of teleworking (holding infection risk and vaccine availability constant).

So far, the optimal share of vaccines given to on-site workers is calculated for different combinations of arbitrary task-specific infection probabilities. Since these parameters are difficult to match with real observations the model is not eligible to make specific proposals on the value of $v_s/V$ for any country. This would not make much sense anyway, because $\beta _r$ and $\beta _s$ may vary depending on the phases of the pandemic and the protective measures imposed. However, previous figures can be used to provide a crude measure that informs us about the need to vaccinate on-site workers in general.

Finally, to get a better resolution on the role of teleworkability in optimal vaccine allocation, we calculate the proportion of $\beta _s$ and $\beta _r$ infection risk combinations where $v_s/V=1$. This measure captures the proportion of epidemiological scenarios when the optimal vaccine allocation strategies strongly prefer on-site workers over remote workers. These proportions are calculated for each industry and for the tradable sector as a whole.

**Figure 4:**
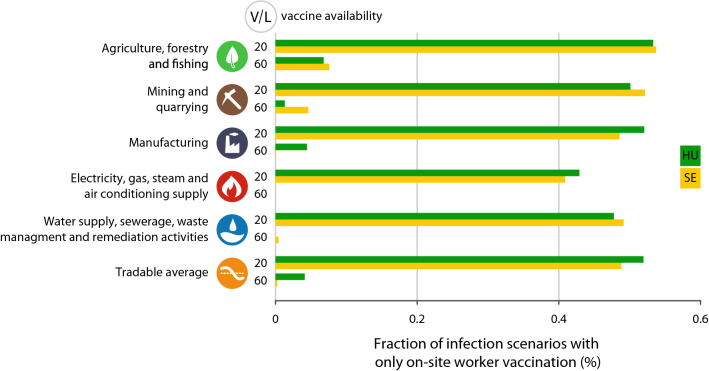
On-site worker vaccination saves jobs in the tradable sector. *V*/*L* denotes vaccine availability for 20–60% of all employees. Percentage values represent the share of infection scenarios when on-site workers shall receive all of the available vaccines to save the most jobs.

Figure 4 presents two cases of vaccine availability, the first is vaccine shortage (only 20% of workers can be vaccinated) and the second is vaccine abundance (the social planner has enough vaccine to immunize 60% of the workers). In cases of vaccine shortage, our results indicate a significant proportion of optimal vaccine distribution scenarios shall concentrate on on-site worker immunization only to minimize unemployment both in Hungary and Sweden. In case of vaccine abundance, the proportion of vaccine distribution scenarios concentrating on on-site workers alone is much lower in both countries. However, these proportions vary by sectors. In the agriculture sector, we still observe scenarios in which vaccinating on-site workers only can be optimal. Nevertheless, sectoral differences can be seen between the countries. While focusing on on-site workers vaccination in the mining sector can be optimal in Sweden, on-site workers immunity is critical in the Hungarian manufacturing industry even with ampleful of available doses.

## Discussion

Vaccinating those who must go out to work even during the most sever phases of the pandemic can save lives by limiting their risk and their ability to further transmit the disease. However, only very few European countries, like Ireland and to some extent Spain^19^, differentiated between occupations in their early vaccination plans (apart from healthcare and social workers). Our paper shows that immunization of on-site workers who can’t work from home can save jobs as well. We build a simple model to show that if teleworkable and non-teleworkable tasks are complements in production, a fall in the supply of on-site workers performing non-teleworkable tasks makes some remote workers redundant in the short-run. Thus, prioritizing on-site workers in vaccination strategies can be economically beneficial for remote workers as well. We show that the optimal share of vaccines allocated to on-site workers depends on short-run infection probabilities and the share of jobs that can be done from home. As long as the vaccine supply is limited and the risk of infection is similar for both worker groups, productivity loss from teleworking should be compensated by vaccinating more remote workers. However, in times of infection waves, when infection risks of on-site workers exceed the risks of remote workers, the optimal allocation favours on-site workers. In this case, after vaccinating the most vulnerable groups and frontline healthcare workers it is advisable to favor those who must commute to work when distributing “residual” vaccines. Later, as more vaccines become available, more advanced economies (e.g. Sweden) should increasingly immunize workers in home-office otherwise there could be redundancies among on-site workers. However, in less developed economies (e.g. Hungary) where the role of teleworkable tasks is smaller in production, the focus should be kept on the vaccination of on-site workers.

Further work is needed to overcome the limitations of the model presented here. We are assuming that economic agents are not adjusting themselves to the dynamically changing circumstances over the pandemic job losses. Although this assumption may be valid in the short run, it is less valid in the case of a prolonged pandemic, when there is enough time for economic actors to adjust to the changed situation. Another limitation of the model is that it considers a single sector and does not take into account input-output relationships between industries. Mass infection of the labour force in a given industry reduces output not only in that particular industry but also in other related industries as well. Extending our basic model in this direction could be used to assess the economic impacts of NPIs^28^, or to tell which industry’s workforce should be vaccinated first in order to keep aggregate unemployment as low as possible. This may depend both on the centrality of the industry in the input-output network and the proportion of occupations that can be done from home.

Finally, there are issues to be resolved regarding the parametrization of the model. Infection risk is incorporated into the model in a very abstract way which does not allow us to relate risk parameters to simulate realistic epidemiological scenarios. Besides, home office productivity losses are assumed to be identical across countries and industries, which is probably not true given the technological and cultural differences that might influence the efficiency of working at home.

## Materials and methods

### Data

For the simulations, we collect pre-Covid employment data on the tradable sector of Sweden and Hungary. For Sweden, we rely on register data from the database ASTRID provided by SCB (Statistics Sweden). This matched employer-employee data covers all workers in the Swedish economy on an annual bases and provides detailed information on the employee and also her employer. Each person is linked to one of 4-digit NACE Rev.2. industries through the characteristics of the employer which allows us to extract employment aggregates for the year 2019. Unfortunatelly, we do not have such a complete database for Hungary so we use large sample survey data instead. More specifically, we use the 2018 wave of the Annual Wage Survey owned by the National Employment Service. This is also a matched employer-employee dataset that covers all employees of all budgetary institutions and a large sample of employees in the private sector. The survey covers all firms employing more than 20 workers, and a random sample of firms employing 20 or less workers. In private firms employing more than 50 workers, the individual data relate to a random sample of the employees while in smaller firms, the data cover all employees (including part-time employees). Like in the ASTRID database, workers are assigned to 4-digit NACE Rev.2. industries.

Although both databases are quite detailed and include 4-digit ISCO occupation codes for each worker, they do not provide direct information on whether occupations are suitable for teleworking in the country concerned. To produce estimates for the potential number of remote and on-site workers ($$L_s $$ and $$ L_r$$) for both countries, we use Dingel and Neiman’s work-from-home classification of which 6-digit SOC (Standard Occupational Classification) occupations can be done from home^10^. Since occupations are coded according to the ISCO (International Standard Classification of Occupations) in the Swedish and Hungarian data, we merge the 6-digit SOC nomenclature with the 4-digit ISCO using the official crosswalk provided by the US Bureau of Labor Statistics. Since the mapping of 6-digit SOC occupations to 4-digit ISCOs is not one-to-one, for some ISCO occupations, determining whether they are suitable for teleworking is not trivial. To address this issue, when more than one SOC occupations can be mapped to an ISCO code, we take the weighted average of the SOC values (0 or 1) using employment counts in the target country as weights. If the resulting value is between 0 and 1, above 0.5 the occupation is considered teleworkable. After merging the work-from-home classification of Dingel and Neiman with the Swedish and Hungarian data, $$L_r$$ and $$L_s$$ is estimated for the whole tradable sector and also its constituent industries. The tradable sector is defined by a selection of non-service NACE Rev.2. industries including agriculture, mining and quarrying, manufacturing, electricity and water supply. Although there are several definitions for the tradable sector, these industries are usually included in most definitions^29,30^.

### Parametrization

All parameters other than task-specific infection probabilities are determined on the basis of real observations. Unit input requirements ($\alpha _r$ and $\alpha _s$) are calibrated so that aggregate employment data fit to the model’s structure. As a first step, $\alpha _r$ is normalized at unity and then $\alpha _s$ is estimated by substituting data on $L_r$ and $L_s$ into Eq. (2). Remote work productivity ($\gamma$) is set to 0.8, a value gathered from a recent firm-level survey on the possibilities of teleworking during the COVID-19 pandemic^27^.

## Data Availability

The ASTRID database is available by permission from Statistics Sweden only. A fee applies. By law, the authors of this paper cannot share the data, interested researchers must contact the data host (Statistics Sweden). The Hungarian annual Wage Survey is available for researchers of the Centre for Economic and Regional Studies (Budapest, Hungary), their co-authors, and students. The work-from-home classification of Dingel and Neiman can be downloaded from the authors’ github page.

## Code availability

Code will be deposited upon acceptance.
